# Perceived Stress and Domain-Specific Physical Activity in Relation to Anxiety and Somatic Symptom Burden Among Romanian Adults: A Cross-Sectional Online Survey

**DOI:** 10.3390/medicina62081530

**Published:** 2026-08-09

**Authors:** Mihaela Fadgyas Stanculete, Octavia Căpățînă, Catalin Alexandru Chihaia

**Affiliations:** 1 Department of Neurosciences, Faculty of Medicine, Iuliu Hațieganu University of Medicine and Pharmacy, 400012 Cluj-Napoca, Romania; mihaela.fadgyas@umfcluj.ro (M.F.S.);; 2 Psychiatry Clinic II, Cluj County Emergency Clinical Hospital, 400012 Cluj-Napoca, Romania

**Keywords:** anxiety, somatic symptoms, perceived stress, physical activity, GPAQ, cross-sectional study, Romania

## Abstract

*Background and Objectives*: Physical activity may relate to mental health differently depending on whether it is performed at work, for transport, or during leisure. Evidence regarding this distinction in Romanian community samples remains limited, and it is unclear whether these associations persist after accounting for perceived stress. We examined domain-specific physical activity, sedentary time and perceived stress in relation to anxiety and somatic symptom burden. *Materials and Methods*: This cross-sectional online survey included 307 Romanian adults aged 18–65 years. Physical activity was measured with the Global Physical Activity Questionnaire, perceived stress with the 14-item Perceived Stress Scale, anxiety with the Beck Anxiety Inventory, and somatic symptoms with the Patient Health Questionnaire-15. Spearman correlations were adjusted for multiple testing with the Benjamini–Hochberg false-discovery-rate procedure. Multivariable linear models used HC3 robust standard errors and included sex, age group, education, body mass index and religious affiliation as covariates. Scores for each physical activity domain were log(1 + x)-transformed and standardized. Sensitivity analyses considered participation versus non-participation, symptom severity categories, total activity and exclusion of an extreme activity value. *Results*: Median scores were 24 for perceived stress, 10 for anxiety and 7 for somatic symptoms. Perceived stress correlated with both anxiety (rho = 0.486) and somatic symptoms (rho = 0.428; both false-discovery-rate-adjusted *p* < 0.001). Vigorous leisure-time activity was inversely correlated with somatic symptoms (rho = −0.205; adjusted *p* = 0.002), whereas total physical activity was not associated with either outcome. In the fully adjusted models, a one-standard-deviation-higher PSS-14 score was associated with 5.57 more BAI points (95% CI 4.50–6.63) and 2.12 more PHQ-15 points (95% CI 1.62–2.63). Vigorous leisure-time activity remained associated with a lower PHQ-15 score (−0.84 points per standard deviation; 95% CI −1.35 to −0.32), but its association with BAI was no longer statistically significant after adjustment for perceived stress. *Conclusions*: Perceived stress showed the strongest association with both anxiety and somatic symptom burden. The use of a single total activity score concealed a more specific inverse association between vigorous leisure-time activity and somatic symptoms. Longitudinal studies using more representative sampling, objective measures of activity and broader confounder assessment are needed to clarify directionality and determine the potential relevance of vigorous leisure-time activity to somatic symptom burden.

## 1. Introduction

Anxiety commonly includes bodily symptoms and often co-occurs with somatic complaints of clinical importance. When these complaints are assessed outside specialist mental-health services, the overlap can complicate diagnosis, increase health-care use and add to functional impairment [1,2]. A biopsychosocial approach therefore needs to consider both perceived stress and modifiable behaviors such as physical activity, which may be related to psychological and somatic symptoms through several shared pathways.

Romania provides a relevant setting for this question. Mental-health stigma remains an important barrier to help-seeking and affects social and occupational life [3]. Studies in Romanian primary care have also reported substantial levels of anxiety and perceived stress, alongside practical difficulties in coordinating general and specialist mental-health services [4,5]. Somatic complaints may consequently be encountered in general medical care before emotional distress is identified. Examining anxiety and somatic symptoms together, with Romanian-language instruments that can be interpreted locally, is therefore clinically useful [2,6].

Although physical activity is generally associated with fewer anxiety symptoms and a lower subsequent risk of anxiety, estimates vary considerably across studies and populations [7]. One reason may be the use of total activity scores, which combine behaviors undertaken in very different settings. Leisure-time exercise is usually chosen by the individual and may provide enjoyment, mastery, social contact and recovery. Activity performed at work may instead be repetitive, externally imposed or physically demanding without offering the same psychological benefits. Reviews of domain-specific activity suggest that these differences in context matter for mental-health outcomes [8,9].

Exercise intensity may add a further distinction. Vigorous activity produces a rapid heartbeat, faster breathing, sweating and muscular strain. In people who fear bodily arousal, these sensations can resemble those experienced during acute anxiety. Encountering them repeatedly during voluntary exercise, in a setting experienced as safe, may reduce anxiety sensitivity through a process similar to interoceptive exposure. In one experimental study, high-intensity aerobic exercise reduced anxiety sensitivity more quickly than low-intensity exercise and produced a greater reduction in fear of anxiety-related bodily sensations [10]. This mechanism cannot be tested with cross-sectional data, but it offers a plausible explanation for an association between vigorous exercise and somatic symptom burden. Effects on sleep, pain processing, autonomic regulation and cardiorespiratory fitness may also be relevant [11].

Perceived stress is closely related to both anxiety and somatic symptoms. Appraising life circumstances as unpredictable or uncontrollable can influence autonomic and hypothalamic–pituitary–adrenal activity, attention to bodily sensations and everyday coping. Stress and physical activity may also influence one another: sustained stress may reduce the time or motivation available for leisure exercise, while regular activity may modify stress reactivity. In a cross-sectional analysis, perceived stress may represent a confounder, an intermediate variable or a consequence of the outcomes under study. Adjustment for PSS-14 can therefore show whether the association with physical activity remains after current stress appraisal is considered, but it cannot establish a direct effect or a mediating pathway. Reverse causation also remains possible, because anxiety and somatic symptoms may increase perceived stress, and physical symptoms may restrict activity.

Previous Romanian studies have documented anxiety and perceived stress in general and primary-care populations [4,5], while evidence on domain-specific physical activity has come mainly from international studies [8,9]. The present study extends this body of literature by examining occupational, transport-related and leisure-time activity separately rather than relying exclusively on total physical activity. It also considers anxiety symptoms and somatic symptom burden within the same participants and examines whether the observed associations persist after perceived stress is included in the models. This distinction is relevant because activities performed in different settings may differ in voluntariness, psychological meaning, social context and opportunities for recovery.

We used data from an online survey of Romanian adults to examine the relationships of perceived stress, domain-specific physical activity and sedentary time with anxiety and somatic symptoms. We first assessed bivariate associations, then estimated adjusted models that included demographic and anthropometric characteristics, and finally examined whether the results were stable under alternative definitions of activity and symptom severity. We hypothesized that vigorous leisure-time activity would be associated with lower anxiety and somatic symptom scores, with greater attenuation of the anxiety association after adjustment for perceived stress. We also expected occupational activity to show no comparable inverse association, in keeping with the proposed mental-health dimension of the physical activity paradox. Perceived stress was expected to have the strongest positive association with both outcomes.

## 2. Methods

### 2.1. Study Design and Participants

We carried out a cross-sectional online survey from 14 April to 3 June 2026. Romanian citizens aged 18–65 years who had internet access were eligible to participate. Exclusion criteria were pregnancy, participation in a competitive sport, a major psychiatric or neurological disorder, and cognitive or physical impairment that substantially limited questionnaire completion or physical activity. Participants were recruited by non-probability snowball sampling. The survey link was circulated through WhatsApp groups and Instagram and was subsequently shared by respondents. Initial dissemination included medical students, physicians, Christian religious communities and their wider social networks. Participation was voluntary, and recruitment was not stratified by sex, age, education, residence or religious affiliation. The recruitment procedure was therefore not designed to generate a sample representative of the Romanian adult population.

A total of 350 questionnaires were submitted. Fifteen respondents met an exclusion criterion: pregnancy (n = 5); competitive sport participation (n = 5); age outside the eligible range (n = 4); or a major psychiatric, neurological, cognitive or physically limiting condition (n = 1). A further 28 questionnaires were excluded because the GPAQ could not be scored consistently. Reasons included missing follow-up responses, non-numeric or ambiguous reports of frequency or duration, and contradictions between participation and duration fields. These records were excluded according to data-consistency criteria because deriving valid domain-specific or total activity scores would have required unverifiable assumptions. The online questionnaire did not prevent all incomplete or internally contradictory GPAQ entries.

The final complete-case sample comprised 307 participants. Recruitment ended on 3 June 2026 because of the project timetable and logistical constraints. The study was not prospectively registered, no formal sample-size calculation was undertaken, and no interim outcome analysis or data-dependent stopping rule was used. A separate manuscript based on the same cohort examines dietary patterns and mental-health outcomes; the two analyses address different exposures and research questions.

### 2.2. Ethics

The study was approved by the Ethics Committee of the Cluj County Emergency Clinical Hospital, Cluj-Napoca, Romania, approval no. 2708/2026. Participation was voluntary and unpaid. Respondents provided electronic informed consent before accessing the questionnaire. The survey was anonymous, and data were handled in accordance with applicable data-protection requirements and the Declaration of Helsinki.

### 2.3. Measures

Physical activity and sedentary time. The Romanian version of the Global Physical Activity Questionnaire (GPAQ) was used to record the frequency and duration of vigorous and moderate activity at work, walking or cycling for transport, vigorous and moderate activity during leisure time, and daily sedentary time [12,13]. Weekly activity was expressed as metabolic-equivalent minutes, using 8 MET for vigorous activity and 4 MET for moderate activity. Domain-specific and total MET-min/week scores were calculated according to the GPAQ analysis guide. The domain scores contained many zero values and had marked right-skewed distributions; they were therefore transformed using log(1 + x) and then standardized. Sedentary hours per day were standardized without transformation.

Perceived stress. The 14-item Perceived Stress Scale (PSS-14) assesses the extent to which recent circumstances are experienced as unpredictable, uncontrollable or overwhelming [14]. We used the Romanian-language version, whose psychometric properties have been studied in a Romanian sample [15]. Scores range from 0 to 56, with higher scores indicating greater perceived stress. Internal consistency in this sample was good (Cronbach’s alpha = 0.838).

Anxiety. The Beck Anxiety Inventory (BAI) consists of 21 items scored from 0 to 3 and gives substantial weight to the subjective and somatic manifestations of anxiety [16]. The Romanian-language adaptation and scoring guidance described by Vraști were used [6]. Total scores ranged from 0 to 63. The primary analyses treated BAI as a continuous measure to retain the information contained in the full score. For descriptive and sensitivity analyses, scores were also grouped as minimal/mild (<22), moderate (22–35) or severe/clinically concerning (>35). Internal consistency was excellent (Cronbach’s alpha = 0.919).

Somatic symptoms. The Patient Health Questionnaire-15 (PHQ-15) measures the burden of 15 common somatic symptoms [17]. We used a Romanian-language version described in Romanian measurement guidance [6]; a general-population adaptation has also been evaluated in Romania [18]. Scores range from 0 to 30. PHQ-15 was analyzed as a continuous measure in the primary models and grouped as minimal (0–4), mild (5–9), moderate (10–14) or severe (15–30) in descriptive and sensitivity analyses. Internal consistency was good (Cronbach’s alpha = 0.832).

Covariates. The questionnaire collected information on sex, age group, place of residence, education, employment, marital and family status, religious affiliation, height and weight. Body mass index (BMI) was calculated as weight in kilograms divided by height in meters squared. The primary multivariable models included sex, age group, education, standardized BMI and religious affiliation. Because several religious categories were small, affiliation was grouped as Orthodox, Protestant/Neo-Protestant, or other/none.

### 2.4. Statistical Analysis

Continuous variables were described using means and standard deviations, medians and interquartile ranges, as appropriate. Categorical variables were reported as counts and percentages. Spearman rank correlations were calculated between the two outcomes and eight exposures: PSS-14, the five physical activity domains, sedentary time and total physical activity. The Benjamini–Hochberg procedure was applied to the 16 correlation *p* values to control the false-discovery rate [19].

Three nested regression models were fitted for each outcome. Model 1 contained the demographic and anthropometric covariates. Model 2 added the five domain-specific activity scores and sedentary time. Model 3 added the standardized PSS-14. Confidence intervals and hypothesis tests were based on HC3 heteroskedasticity-consistent standard errors [20]. For continuous predictors, each coefficient is the adjusted mean difference in the raw BAI or PHQ-15 score associated with a one-standard-deviation difference in the predictor.

Sensitivity analyses recoded each activity domain as participation versus non-participation, replaced the domain scores with total GPAQ activity, and repeated the models after excluding one respondent whose reported activity exceeded 960 min per day across domains. We also fitted exploratory proportional-odds models for the BAI and PHQ-15 severity categories and tested a sex-by-stress interaction. Approximate parallel-lines diagnostics did not support the proportional-odds assumption for the full ordinal models, so those estimates were retained only as exploratory results. Available demographic and symptom characteristics were also compared descriptively between participants included in the analytical sample and those excluded because valid GPAQ scores could not be derived. Analyses were conducted in Python 3 with pandas 2.2.3, SciPy 1.17.0 and statsmodels 0.14.6. Tests were two-sided, with *p* < 0.05 used as the nominal significance threshold; multiplicity adjustment was applied to the defined family of correlations. Because the study and analysis plan were not prospectively registered, the analyses were interpreted as hypothesis-informed rather than strictly confirmatory. The proportional-odds models and the sex-by-perceived-stress interaction were considered exploratory. The manuscript was prepared in accordance with the STROBE statement [21].

## 3. Results

### 3.1. Participant Characteristics

The final sample comprised 307 participants. Women accounted for 79.2% (n = 243), 78.8% (n = 242) lived in urban areas, and half of the sample was aged 25–34 years (n = 154, 50.2%). Most participants had completed or were enrolled in higher education. Protestant/Neo-Protestant respondents represented 53.7% of the sample and Orthodox respondents 38.4%, a distribution that reflected the initial recruitment channels rather than the religious composition of the Romanian population. Mean BMI was 23.22 kg/m^2^ (SD 4.67). Based on the predefined BAI severity categories, 254 participants (82.7%) had minimal or mild anxiety symptoms, 39 (12.7%) had moderate symptoms, and 14 (4.6%) had severe or clinically concerning symptoms. Participants excluded because valid GPAQ scores could not be derived were broadly similar to the analytical sample in the proportions of women (85.7% vs. 79.2%), urban residents (82.1% vs. 78.8%) and Protestant/Neo-Protestant respondents (50.0% vs. 53.7%). Their median symptom scores were descriptively higher: 13.5 versus 10 for BAI, 26.5 vs. 24 for PSS-14, and 10 versus 7 for PHQ-15 (Table 1).

**Table 1:** Participant characteristics (N = 307).

Characteristic	Value
Women	243 (79.2%)
Men	64 (20.8%)
Age 18–24 years	65 (21.2%)
Age 25–34 years	154 (50.2%)
Age 35–44 years	53 (17.3%)
Age 45–54 years	25 (8.1%)
Age 55–65 years	10 (3.3%)
Urban residence	242 (78.8%)
Rural residence	65 (21.2%)
High school/vocational education	32 (10.4%)
Bachelor’s level	149 (48.5%)
Master’s level	116 (37.8%)
Doctorate level	10 (3.3%)
Employed	238 (77.5%)
Student	60 (19.5%)
Other employment status	9 (2.9%)
Orthodox	118 (38.4%)
Protestant/Neo-Protestant	165 (53.7%)
Other/none	24 (7.8%)
BMI, kg/m^2^	23.22 (4.67); median 22.41 (20.08–25.30)
PSS-14 score	24.03 (7.37); median 24 (19–28)
BAI score	12.91 (10.51); median 10 (5–19)
BAI severity: minimal/mild/moderate/severe or clinically concerning	254 (82.7%)/39 (12.7%)/14 (4.6%)
PHQ-15 score	7.65 (4.97); median 7 (4–11)
Total physical activity, MET-min/week	3778.51; median 2160 (840–4800)
Sedentary time, hours/day	7.18 (3.26); median 7 (5–10)
Low/moderate/high GPAQ level	57 (18.6%)/131 (42.7%)/119 (38.8%)

Note. Values are n (%), mean (SD), or median [interquartile range]. BMI, body mass index; BAI, Beck Anxiety Inventory; GPAQ, Global Physical Activity Questionnaire; MET, metabolic equivalent; PHQ-15, Patient Health Questionnaire-15; PSS-14, 14-item Perceived Stress Scale. BAI severity categories were defined as minimal/mild (<22), moderate (22–35), and severe/clinically concerning (>35).

### 3.2. Bivariate Associations

Perceived stress was positively correlated with both anxiety and somatic symptoms. After false-discovery-rate correction, vigorous leisure-time activity was inversely correlated with the PHQ-15 score. Two other correlations fell just above the adjusted threshold: vigorous leisure-time activity with BAI (rho = −0.141; unadjusted *p* = 0.014) and moderate occupational activity with BAI (rho = 0.137; unadjusted *p* = 0.016); both had q = 0.053. No association with either outcome was found for total GPAQ activity, active transport, moderate leisure-time activity or sedentary time (Table 2).

**Table 2:** Spearman correlations of perceived stress and physical activity with symptom scores.

Exposure	BAI rho	*p*	FDR q	PHQ-15 rho	*p*	FDR q
Perceived stress (PSS-14)	0.486	<0.001	<0.001	0.428	<0.001	<0.001
Occupational vigorous PA	−0.040	0.481	0.744	0.004	0.943	0.943
Occupational moderate PA	0.137	0.016	0.053	0.086	0.132	0.351
Active transport	0.030	0.597	0.744	0.030	0.604	0.744
Leisure-time vigorous PA	−0.141	0.014	0.053	−0.205	<0.001	0.002
Leisure-time moderate PA	−0.021	0.716	0.818	−0.041	0.478	0.744
Sedentary time	−0.065	0.258	0.515	−0.069	0.225	0.514
Total physical activity	0.011	0.842	0.898	−0.032	0.580	0.744

Note. The Benjamini–Hochberg procedure was applied across the 16 exposure–outcome correlations. PA, physical activity.

### 3.3. Multivariable Models

After adjustment for demographic characteristics, BMI and all activity domains, vigorous leisure-time activity was associated with lower BAI (Table 3) and PHQ-15 (Table 4) scores. The proportion of explained variance rose from 9.7% to 35.0% for BAI and from 14.1% to 30.5% for PHQ-15 when perceived stress was added. The corresponding increases in R2 (approximately 0.25 and 0.16) were larger than the contribution of the activity variables considered together. In the fully adjusted BAI model, a one-standard-deviation-higher PSS-14 score was associated with 5.57 more BAI points (95% CI 4.50–6.63). The coefficient for vigorous leisure-time activity decreased from −1.88 to −1.02 and was no longer statistically significant (*p* = 0.086). In the PHQ-15 model, both perceived stress and vigorous leisure-time activity remained associated with somatic symptom burden.

**Table 3:** Adjusted associations with BAI score.

Exposure	Model 2 B (95% CI)	*p*	Model 3 B (95% CI)	*p*
Occupational vigorous PA	0.00 (−1.72 to 1.73)	0.996	0.29 (−0.96 to 1.55)	0.646
Occupational moderate PA	1.12 (−0.19 to 2.43)	0.093	0.97 (−0.20 to 2.14)	0.105
Active transport	−0.14 (−1.39 to 1.11)	0.829	−0.13 (−1.19 to 0.94)	0.816
Leisure-time vigorous PA	−1.88 (−3.19 to −0.57)	0.005	−1.02 (−2.19 to 0.14)	0.086
Leisure-time moderate PA	0.23 (−1.21 to 1.67)	0.755	0.26 (−0.95 to 1.47)	0.672
Sedentary time	−1.05 (−2.27 to 0.17)	0.092	−1.06 (−2.18 to 0.06)	0.064
Perceived stress (PSS-14)	Not included		5.57 (4.50 to 6.63)	<0.001
Model R2/adjusted R2	0.097/0.044		0.350/0.309	

Note. Model 2 was adjusted for sex, age group, education, standardized BMI, religious affiliation, all five physical activity domains and sedentary time. Model 3 additionally included standardized PSS-14. Activity variables were log(1 + x)-transformed and standardized; sedentary time and PSS-14 were standardized. HC3 robust standard errors were used.

**Table 4:** Adjusted associations with PHQ-15 score.

Exposure	Model 2 B (95% CI)	*p*	Model 3 B (95% CI)	*p*
Occupational vigorous PA	0.28 (−0.44 to 0.99)	0.448	0.39 (−0.24 to 1.01)	0.230
Occupational moderate PA	0.12 (−0.49 to 0.74)	0.694	0.07 (−0.52 to 0.65)	0.824
Active transport	−0.23 (−0.81 to 0.36)	0.449	−0.22 (−0.75 to 0.31)	0.412
Leisure-time vigorous PA	−1.16 (−1.72 to −0.61)	<0.001	−0.84 (−1.35 to −0.32)	0.001
Leisure-time moderate PA	0.11 (−0.52 to 0.74)	0.735	0.12 (−0.43 to 0.67)	0.667
Sedentary time	−0.29 (−0.87 to 0.29)	0.331	−0.29 (−0.87 to 0.28)	0.318
Perceived stress (PSS-14)	Not included		2.12 (1.62 to 2.63)	<0.001
Model R2/adjusted R2	0.141/0.090		0.305/0.262	

Note. Model definitions and scaling are identical to Table 3. HC3 robust standard errors were used.

### 3.4. Sensitivity Analyses

**Figure 1:**
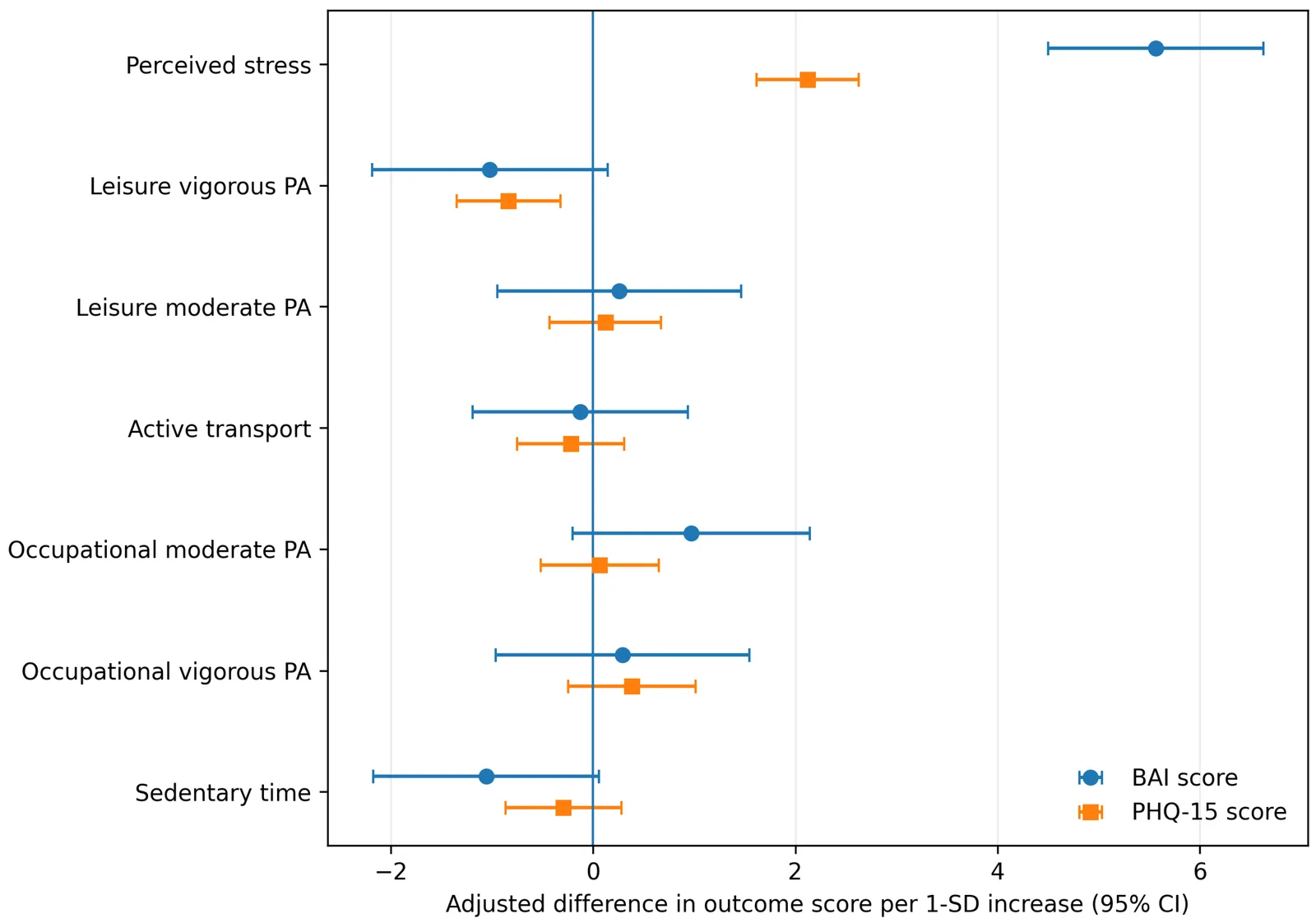
Fully adjusted associations of perceived stress and domain-specific physical activity with BAI and PHQ-15 scores. Points show regression coefficients and bars show 95% confidence intervals. Models included sex, age group, education, standardized BMI, religious affiliation, all displayed activity domains and perceived stress. PA, physical activity.

The main pattern was unchanged when vigorous leisure-time activity was coded as any participation versus none. Before adjustment for perceived stress, participation was associated with BAI scores that were 3.83 points lower (95% CI −6.62 to −1.04) and PHQ-15 scores that were 2.41 points lower (95% CI −3.60 to −1.21). After PSS-14 was added, the BAI difference was smaller and no longer statistically significant (−1.94; 95% CI −4.41 to 0.53), whereas the PHQ-15 difference remained (−1.69; 95% CI −2.80 to −0.57). In the exploratory proportional-odds models that included stress, the odds ratio was 0.46 (95% CI 0.28–0.78) for PHQ-15 severity and 0.85 (95% CI 0.35–2.02) for BAI severity (Figure 1). Because the proportional-odds assumption was not supported for the full models, these estimates were not used in the primary interpretation.

Excluding the respondent who reported more than 960 min of activity per day produced a similar coefficient for vigorous leisure-time activity in the PHQ-15 model (−0.83; *p* = 0.002). The corresponding BAI coefficient remained close to the significance threshold (−1.13; *p* = 0.052). Total GPAQ activity was not associated with either outcome, with or without adjustment for stress. There was no evidence of a sex-by-PSS interaction for BAI (*p* = 0.156) or PHQ-15 (*p* = 0.388).

## 4. Discussion

### 4.1. Principal Findings

The findings were consistent with the hypothesis regarding somatic symptom burden and provided partial support for the hypothesis regarding anxiety. Perceived stress accounted for the largest share of explained variation in anxiety and somatic symptom scores. Vigorous leisure-time activity, rather than total activity, was associated with fewer somatic symptoms. It was also associated with lower anxiety before perceived stress was added to the model, but this association was attenuated after adjustment. Thus, the observed pattern was consistent with the first hypothesis for somatic symptom burden but only partly consistent with it for anxiety. Neither vigorous nor moderate occupational activity showed a comparable inverse association with either outcome, as anticipated in the second hypothesis.

### 4.2. Interpretation in Relation to Previous Research

The magnitude of the stress associations is consistent with the content of the measures. PSS-14 captures the appraisal of current demands, while BAI and PHQ-15 include symptoms that may accompany sustained autonomic arousal, poor sleep and increased attention to bodily sensations. These cross-sectional data, however, cannot establish the direction of the relationship. Anxiety and somatic distress may themselves raise perceived stress, and unmeasured adversity could influence stress, activity and symptoms at the same time. The reduction in the activity coefficient after PSS-14 was introduced should therefore be understood as attenuation within the model, not as evidence of mediation.

The difference between leisure-time and total activity accords with systematic reviews showing that mental-health associations vary by activity domain [8,9]. Prospective cohort studies have likewise reported different patterns for recreational and occupational activity in relation to incident anxiety and related symptoms [22,23]. The total GPAQ score combines voluntary exercise, occupational exertion and active transport, even though these activities differ in motivation, social setting and opportunities for recovery. Combining them may therefore dilute an association that is confined to a particular domain. The recent multilevel meta-analysis reached a similar conclusion, while also noting substantial heterogeneity between studies [9].

Several mechanisms could account for the association with PHQ-15. Vigorous exercise produces tachycardia, sweating, rapid breathing and muscular strain, all of which can be perceived as threatening by people with high anxiety sensitivity. Repeatedly experiencing these sensations during chosen, controlled activity may reduce fear of bodily arousal and the tendency to interpret somatic sensations catastrophically. Experimental work showing a reduction in anxiety sensitivity after high-intensity aerobic exercise supports this interpretation [10]. Better cardiorespiratory fitness, sleep and pain regulation may also contribute. The association with BAI, by contrast, weakened after current stress appraisal was included, suggesting that stress was more closely related to anxiety symptoms in this sample. Selection remains an equally plausible explanation: people with a high somatic symptom burden may be less able or less willing to undertake vigorous leisure exercise.

Sedentary time was not positively associated with either outcome. Measurement error may have contributed, because a single self-reported estimate of daily sitting does not distinguish work from leisure, prolonged sitting from frequently interrupted sitting, or screen time from other sedentary activities. The age and educational profile of the sample may also have limited variability. Studies using accelerometry together with repeated information on context and posture would provide a more informative test of the relationship between sedentary behavior and symptom burden.

### 4.3. Strengths and Limitations

This study has several methodological strengths. Anxiety and somatic symptoms were assessed in the same participants, the three psychometric scales showed good to excellent internal consistency, and physical activity was separated by domain rather than summarized only as a total score. The analysis also used false-discovery-rate correction for the correlation family, HC3 robust standard errors and several sensitivity checks.

Several limitations should be considered. First, the cross-sectional design does not permit conclusions about temporal sequence or causation. Reverse causation remains plausible, as participants with a higher somatic symptom burden may have been less able or less willing to engage in vigorous leisure-time activity.

Second, non-probability snowball recruitment produced a self-selected and non-representative sample with a high proportion of women, adults aged 25–34 years, urban residents, university-educated participants and members of Protestant/Neo-Protestant communities. This composition likely reflects the initial dissemination of the survey through medical, academic and Christian community networks; its subsequent circulation through participants’ personal networks; and the online, voluntary recruitment format. These factors limit the generalizability of the findings beyond the present sample. The religious composition differed markedly from that of the wider Romanian population and may have been associated with differences in lifestyle, social support, community participation or religious coping. Although broad religious affiliation was included as a covariate, this adjustment could not account for the intensity of religious practice, coping style, community support or related lifestyle behaviors.

Third, all measures were self-reported, creating the possibility of recall bias, reporting bias and shared-method variance. GPAQ is particularly susceptible to inaccurate recall and over-reporting. In addition, BAI and PHQ-15 quantify anxiety symptoms and somatic symptom burden, respectively, but do not establish clinical diagnoses.

Fourth, the exclusion of 28 participants because valid GPAQ scores could not be derived may have introduced additional selection bias. Although their demographic composition was broadly similar to that of the analytical sample, their median BAI, PSS-14 and PHQ-15 scores were descriptively higher. The direction and magnitude of any resulting selection bias cannot be determined from the present data. Stronger automated range restrictions and cross-field logic checks should be incorporated into future online applications of the instrument.

Fifth, the dataset lacked several potentially important confounders, including sleep quality, chronic medical illness, pain diagnoses, medication use, smoking, alcohol intake, income and objectively measured fitness. Residual confounding therefore remains possible, and the adjusted coefficients should not be interpreted as causal or fully independent effects.

Sixth, only 14 participants (4.6%) had severe or clinically concerning anxiety symptoms. This sparse upper category limited the stability and interpretability of the ordinal analyses and prevented meaningful subgroup comparisons across the full range of anxiety severity. The primary interpretation was therefore based on BAI as a continuous outcome, while the ordinal models were retained as exploratory sensitivity analyses.

Finally, the study was not prospectively registered. The hypotheses and analyses should therefore be viewed as hypothesis-informed rather than strictly confirmatory.

### 4.4. Implications

These results have implications mainly for measurement and future study design. Physical activity should be examined by domain, because a total MET-min/week score may conceal associations that are limited to leisure exercise. Prospective work should combine accelerometry with information on the setting and purpose of the activity, repeated measures of perceived stress, and clinical assessment of somatic symptoms. The present data are not sufficient to recommend vigorous exercise as treatment. They did, however, identify a potentially informative sample-level association: after adjustment for PSS-14, participants who reported any vigorous leisure-time activity had PHQ-15 scores that were, on average, 1.69 points lower than those of non-participants (95% CI −2.80 to −0.57). This difference should not be interpreted as an effect of vigorous activity, because reverse causation, selection bias and residual confounding remain plausible. In future research and exploratory clinical assessment, distinguishing vigorous leisure-time activity from total physical activity may provide more contextual information than relying exclusively on a total activity score. Any advice about exercise must still take account of medical suitability, safety and personal preference, and causal recommendations require longitudinal or interventional evidence.

## 5. Conclusions

In this non-probability online sample of Romanian adults, perceived stress was positively associated with both anxiety symptoms and somatic symptom burden. Self-reported vigorous leisure-time activity was inversely associated with somatic symptom burden after covariate adjustment, whereas total physical activity was not associated with either outcome. Given the cross-sectional design, self-selected sample, exclusive reliance on self-report, and potential for residual confounding and reverse causation, these findings should not be interpreted as evidence of a protective or therapeutic effect. They support the inclusion of the activity domain and context in future longitudinal studies using more representative sampling, objective activity measures and broader assessments of potential confounders.

## Data Availability

De-identified data and analysis code may be made available by the corresponding author on reasonable request, subject to the conditions of the ethics approval and institutional data-sharing requirements.
